# Anomalous aortic origin of coronary arteries from the opposite sinus: A critical appraisal of risk

**DOI:** 10.1186/1471-2261-12-83

**Published:** 2012-10-01

**Authors:** Josiah M Peñalver, Ralph S Mosca, Daniel Weitz, Colin KL Phoon

**Affiliations:** 1 Division of Pediatric Cardiology, Department of Pediatrics, 160 East 32nd Street, L-3, New York, NY 10016, USA; 2 Division of Pediatric and Adult Congenital Cardiac Surgery, Department of Cardiothoracic Surgery, 530 First Avenue, Suite 9-V, New York, NY 10016, USA; 3 Leon H. Charney Division of Cardiology, Department of Medicine, New York University School of Medicine, 550 First Avenue, New York, NY 10016, USA

**Keywords:** Coronary artery anomaly, Sudden cardiac death, Anomalous aortic origin of the coronary artery, Heart defects, Congenital

## Abstract

**Background:**

Anomalous aortic origin of the coronary artery (AAOCA) from the opposite sinus of Valsalva with an interarterial course has received much attention due to its association with sudden death in otherwise healthy individuals. AAOCA is relatively common and may have significant public health implications. While our knowledge of its pathophysiology and natural history remains incomplete, an emphasis has been placed on surgical correction.

**Discussion:**

In 2005 we published a review examining the rates of sudden death with AAOCA, as well as complications of surgical management. Evidence now points even more strongly to lower rates of sudden death, while surgical outcomes data now better documents associated risks.

**Summary:**

Armed with this updated information, we agree with the need for a national registry to better track patients with AAOCA. We submit that the risks of surgical management outweigh any benefits in the asymptomatic patient with anomalous right coronary artery, and expectant management should also be strongly considered even in asymptomatic patients with anomalous left coronary artery.

## Background

Anomalous aortic origin of the coronary artery arising from the opposite sinus (AAOCA) has received much attention due to its association with sudden cardiac death (SCD). It is believed to be relatively common and is thus a potentially serious public health hazard. Indeed, many groups opt for surgical “repair” of AAOCA when the coronary artery takes an intramural and/or interarterial course even if a patient is asymptomatic. In a critical analysis in 2005, we concluded that the intrinsic risk of AAOCA was far lower than perceived and that surgical risks were not insignificant [1]. Since that time, important data have emerged on both the rates of SCD in otherwise healthy individuals with AAOCA and surgical outcomes. The goals of this paper are three-fold: 1) to review briefly the diagnosis and presumed pathophysiology of AAOCA; 2) to show recent data supporting our argument that the risk of SCD is far lower than many believe; and 3) to analyze recent surgical data, which cast serious questions as to whether this is the best management option.

## Discussion

### Pathophysiology

The history of AAOCA has been extensively reviewed. Cheitlin et al. in their 1974 paper demonstrated that an anomalous left coronary artery arising from the right sinus of Valsalva (ALCA) can cause SCD [2]. Others have reported that an anomalous right coronary artery arising from the left sinus of Valsalva (ARCA) can also lead to SCD and have concluded that it too should be considered a potentially dangerous anomaly [3-5].

Several theories have been developed to explain the mechanism of SCD in patients with AAOCA. One theory focuses on the acute angle of takeoff and resultant slit-like orifice and kinking of the anomalous vessel [3,5-7] which, during exercise, may occlude as the aorta expands, leading to an acute ischemic event [2]. Others postulate that increased flow through the aorta and the pulmonary artery during exertion can compress the coronary artery thus obstructing flow [2,3]. Additionally, Basso and her colleagues state that ischemia likely occurs in “infrequent bursts” which lead to myocardial fibrosis and subsequent foci for lethal ventricular arrhythmias. This theory is supported by histologic evidence of chronic ischemic changes in areas of myocardium supplied by the anomalous coronary vessels [8,9]. Intravascular ultrasound (IVUS) has demonstrated that intussuception, coronary hypoplasia and lateral compression of the coronary wall by the aorta may contribute to compromised perfusion [10,11]. We also believe restriction of flow through the relatively noncompliant “pericommissural” area may also be a factor. It is theorized that any of these mechanisms individually or in the aggregate could be potentiated by changes in blood flow during exercise and could thereby lead to SCD.

### Diagnosis

Patients with AAOCA are typically asymptomatic. The diagnosis is often made as an incidental finding. Symptomatic patients complain of exertional syncope, chest pain, or palpitations [8]. The physical exam, ECG and exercise stress testing are generally unremarkable [8,12]. Because coronary angiography has a significant false negative rate [13,14], other imaging modalities are frequently employed. In particular, multidetector computed tomography (CT) scanners and magnetic resonance angiography (MRA) now provide excellent spatial resolution allowing visualization of the coronary anatomy [15-18] and have been used to diagnose AAOCA in patients who had previously “normal” angiograms [19,20]. One study demonstrated that gadolinium-enhanced MRA in adults can be used to assess scar burden in the area supplied by an anomalous right coronary artery, which may have prognostic value in the future [21]. Advances in CT technology have reduced the radiation exposure and ameliorated the need for heart rate control with beta-blockers [20]. In the pediatric population, even with lower radiation doses, the risk of exposure limits the usefulness of CT. The American Heart Association in its recent statement on noninvasive imaging of coronary arteries in the young recommends MRA over angiography and CT in assessing for anomalous coronary arteries [22].

In children, transthoracic echocardiography (TTE) is arguably the most useful tool in screening for this condition and often leads to the diagnosis without additional imaging [23-26]. TTE is non-invasive, does not employ radiation and can be performed without sedation. It allows for direct visualization of the origins of the vessels [27] and when aided by the use of color Doppler can demonstrate an intramural course that might otherwise be missed [28]. Clearly, as technology improves the value of any single modality in assessing this condition may change. We, like many groups, have often correctly diagnosed this condition using TTE but prefer additional supplemental imaging modalities both to confirm this important diagnosis and to delineate specific details of the anatomy.

### Prevalence

The true prevalence of this condition is difficult to ascertain. Published data suggests that the rate of anomalous coronary arteries arising from the opposite sinus to be in the range of 0.1%-0.3% [23,24,29,30]. In one prospective angiographic study involving 1,950 patients by Angelini et al., the authors found the incidence to be as high as 1.07% [31]. For the purposes of this paper we will use a conservative estimate of 0.1-0.2%. ARCA is estimated to be six to ten times more common then ALCA [29,31].

### Risk of sudden death with AAOCA

In this section we will update estimates of the risk of SCD with AAOCA and challenge the assumption that the risk of dying suddenly is high, as continues to be cited by many authors [18,32-35].

Estimates on rates of SCD with this condition come almost exclusively from autopsy data. Based on several autopsy studies, mortality “rates” have been reported to range between 0%-50% with ARCA and 30%-100% with ALCA [2,4,5,7,36]. The results of several often-quoted autopsy papers are summarized in Table 1. These numbers are inherently biased by the selected population: the deceased. The numbers reported reflect the prevalence of AAOCA in those who have already died, *not* the risk of death of those living with anomalous coronary vessels. This is akin to arguing that the risk of suicide in American males age 15-24-years-old is 12% because of the 35000 deaths annually in this age group, 4200 were from suicide [37]. Clearly (and thankfully), 1 in 8 males in this age group do not commit suicide.

**Table 1:** Five representative autopsy studies (adapted from Mirchandani and Phoon, 2005)

**Author**	**N**	**ARCA**	**ALCA**	**Sudden deaths attributed to anomalous coronaries**	
				**Right**	**Left**
Chietlin et al., 1974 [2]	51	18	33	0/18 (0%)	9/33 (27%)
Taylor et al., 1992 [7]	30	21	9	4/21 (19%)	8/9 (89%)
Kragel and Roberts, 1988 [36]	32	25	7	8/25 (32%)	5/7 (71%)
Taylor et al., 1997 [4]	101	52	49	13/52 (25%)	28/49 (57%)
Frescura et al., 1998 [5]	11	7	4	4/7 (57%)	4/4 (100%)
TOTAL	225	123	102	29/123 (24%)	54/102 (53%)

Abbreviations: ARCA = Anomalous right coronary artery from the left sinus; ALCA = Anomalous left coronary artery from the right sinus.

The actual risk of dying from either ARCA or ALCA must be far lower if one is to believe the prevalence of the disease in the general population. As reported above, prospective screening data estimates 0.1-0.2% of the population have this anomaly. The current US population is ~309 million [38]. Thus, an estimated 300,000-600,000 people currently live in the United States with AAOCA. Further, the annual birth rate in the United States is ~4.3 million [39] suggesting that there are approximately 4000–8000 infants born with AAOCA every year. Clearly, if children and young adults were dying at the rates predicted by autopsy reports, SCD in the young would be a much more common event.

Several studies have indicated that risk of death with this lesion is far lower than what has often been cited based on autopsy studies. In 2000, Wren, O’Sullivan and Wright [40] published on all deaths at age 1–20 over a 10-year period in the Northern Health Region of England. The reported population was nearly 800,000 and over the study period provided just over eight million person-years. During that time there were 270 sudden deaths, none of which was attributed to AAOCA. Of note, 41 of these deaths remained unexplained despite necropsy. Using a conservative estimate of 0.1-0.2%, one would expect approximately 800–1,600 individuals living with AAOCA in the region. This study did not evaluate deaths in those older than 20, though certainly such individuals with AAOCA are still at risk for SCD. Additionally, the cause of 41 deaths remains uncertain.

Eckart et al. studied death rates in military recruits during boot camp over a 25-year period [41]. The authors looked at all non-traumatic deaths with available autopsy data. From a population of greater than 6 million military personnel, 21 deaths were associated with AAOCA; all were ALCA. The incidence of SCD attributable to anomalous coronary arteries in the studied population was ~ 1/300,000 (0.0003%). Reasonably, 0.1-0.2% of this population would be expected to have AAOCA – or approximately 6,000-12,000 of the military recruits. Therefore, the risk of death with this condition could be estimated to be 0.17-0.35% (21/6,000-12,000) in individuals engaged in frequent vigorous exercise. Of note, this data was collected during a short period of time in each individual’s life and as such it does not account for death that occurs at a later time.

Corrado and his group evaluated cardiovascular causes of sudden death in young athletes in the Veneto region of Italy from 1979 to 2004 [42]. This study, which was designed primarily to assess the effectiveness of preparticipation screening, elucidates the risk of sudden cardiac death from AAOCA. The Veneto region is a homogenous, geographically well-defined region in which all fatalities in people 35 years or younger are investigated. The authors focused on all sudden cardiovascular deaths in people age 12–35 years old during the 26-year study period. They reported that during that time, the rate of death from congenital coronary anomalies was 0.24 per 100,000 person years. This number includes deaths from *all* forms of congenital coronary anomalies and therefore is higher than the rate of death specifically from AAOCA. Regardless, the number is far lower than that reported by autopsy data.

Maron and his colleagues provide a comprehensive analysis of sudden deaths among competitive athletes in the United States over a 27-year period [43]. The authors used several methods to identify these events. These included: 1. LexisNexis archives; 2. News accounts; 3. Internet searches; 4. Reports from the US Consumer Product Safety Commission; 5. Records from the National Center for Catastrophic Sports Injury Research; 6. Pathology archives; 7. Direct reporting. There were 1866 episodes of sudden death among U.S. athletes (which includes 85 survivors of cardiac arrest) over the 27 years. Of these, 1049 were attributed to a cardiovascular cause and 119 (~11% of the cardiovascular deaths) were attributed specifically to AAOCA. This study is more comprehensive than that of Eckart’s group in that it looks at rates of SCD over an extended period of time. Those athletes who continued to involve themselves in sports remained in this study. Based on their data, the authors report the incidence of sudden cardiovascular mortality 0.61/100,000 person-years (of which anomalous coronaries account for ~11% or 0.07/100,000 person-years). A further analysis was conducted by Brothers and her group using this same data and they determined that the cumulative risk of death over a 20-year period from the age of 15–35 (the highest risk period) in patients with AAOCA was 6.3% for ALCA and 0.2% for ARCA [44]. These analyses are prone to ascertainment bias and underreporting, and may therefore underestimate the true risk of SCD. But, the risk of death with AAOCA remains far lower than what is often cited.

A comprehensive study was conducted by Harmon et al. reviewing SCD in National Collegiate Athletic Association (NCAA) student-athletes from 2004–2008 [45]. The advantages of this study over Maron’s was the ability of the authors to more precisely determine the number of athletes involved in college athletics during the study period. While autopsy data and therefore specific diagnosis were not provided, the authors did find a nearly four-fold higher rate of SCD when compared to Maron’s results. Interpreting this data is difficult without autopsy results, but estimating that ~10% of those episodes of SCD may have been due to AAOCA (based on Maron’s findings), the risk of SCD remains low (~1:430,000 person-years). Note that this is the risk of *all* student-athletes dying from AAOCA from the opposite sinus. If we assume a 0.2% prevalence of AAOCA in the general population, then the risk if a student-athlete actually has an AAOCA from the opposite sinus becomes 1:860 person-years (0.12%); this number is remarkably consistent with other estimates presented in this paper. Additional smaller studies have reported on SCD rates in various populations and lend further support the findings of Wren, Corrado, Eckart, Maron and Harmon. These studies are summarized in Table 2.

**Table 2:** Reports on rates of sudden death

**Author**	**Population studied**	**Total population (n)**	**Study period**	**Total sudden deaths**	**Cardiac related deaths**	**Deaths due to confirmed coronary anomalies**
Wren et al., 2000 [40]	All children 1-20yo in Northern Health Region, England	806,000	1985-1994 (10 years)	270	26	0
Eckart et al., 2004 [41]	All US military recruits	6,300,000	1977-2001 (25 years)	126	64	21
Corrado et al., 2006 [42]	All people 12–35 years old in Veneto Region, Italy	4,379,900	1979-2004 (26 years)	N/A^‡^	320	21
Redelmeier and Greenwald, 2007 [46]	Marathoners from 26 selected US marathons	3,292,268	1975-2004 (30 years)	26	21	2
Maron et al., 2009 [43]	All competitive US athletes	Not available	1980-2006 (27 years)	1866	1049	119
Chugh et al., 2009 [47]	All children 0-17yo in Multnomah County, OR	660,486^*^	2002-2005 (3 years)	8	3	0
Harris et al., 2010 [48]	All triathletes in USA Triathalon sanctioned events	959,214	2006-2008 (3 years)	14	7^†^	1^†^
Harmon et al., 2011 [45]	All NCAA athletes	393,932^§^	2004-2008 (5 years)	80	45	N/A^§^

^*^ Total population of Multnomah County, OR including children and adults.

^†^ Officially listed cause of death “drowning” (during a triathalon event) but cardiac abnormalities were identified and thought to be the causative factor.

^‡^ Study only looked at cardiovascular causes of sudden death.

^§^ Total population derived by dividing “athelete participation years” by the 5-year study period. The study did not report on specific causes of cardiac death.

Both Eckart’s and Maron’s studies focused on populations (military recruits and athletes) that are by definition involved in a high level of physical activity. These rates of SCD are likely higher than are to be expected for the US population in general since only the minority of U.S. residents meets minimum exercise requirements [49,50]. In actuality, there are approximately 3.7 million competitive athletes involved in high-risk activities annually [51], representing only a fraction of the total US population.

Physical exertion is thought to significantly increase risk of SCD based on the reported cases and the presumed pathophysiology. Those not involved in competitive sports or physically demanding jobs are at lower risk of SCD. In 2000, Basso and her colleagues published a review of all sudden deaths reported in a large US and Italian registry. 27 deaths were attributed solely to anomalous coronary arteries (23 due to ALCA, 4 due to ARCA). All the deaths occurred during or immediately after strenuous athletic activity [8]. Corrado and his group, from the Veneto region of Italy as discussed above, demonstrated that the risk of sudden cardiac death from coronary anomalies is increased 3 to 6 fold with athletic activity [42]. Thiene et al. reported a threefold increase in SCD (from all causes) among athletes when compared to non-athletes [9]. The risk of SCD reported in Eckart’s and Maron’s studies may therefore be high relative to the general population.

### Is risk stratification of patients possible?

AAOCA receives a disproportionate amount of media attention because of its association with SCD in otherwise healthy and often asymptomatic individuals. Identifying specific attributes associated with those who die from this condition would help with risk stratification and prognosis. Several authors have attempted to identify specific factors that might portend a higher likelihood of SCD. Anatomic characteristics that have been discussed as possibly contributing to the risk of death include those features already listed above, namely: angle of takeoff; intramural course; slit-like ostium; interarterial course; vessel spasm and intussusception of the anomalous vessel [10,12]. In an attempt to identify specific features that were thought to contribute to risk of death, Taylor et al. looked at 30 pathology cases of anomalous left and right coronary arteries. The authors were unable to identify any specific anatomical features that correlated with an increase risk of demise. The authors concluded that there were no anatomic features that could aid in risk assessment [4].

Based on their evaluation using IVUS in symptomatic patients with ALCA, Angelini and his colleagues have postulated that a subset of patients with intussusception of the anomalous coronary are at high risk for SCD [11,52,53]. The authors were able to define three novel features that correlate with clinical severity: degree of hypoplasia of the intramural segment; amount of lateral compression of intussuscepted segment; degree of lateral compression during periods of exertion. Ultimately they hope to use their findings to facilitate clinical decision-making. Its use in children may be limited due to the invasive nature of cardiac catheterization and IVUS, lack of experience using this technique in children leading to increased procedural risk, and limitations in catheter size for use in children.

The only variable that seems to consistently correlate with risk of SCD is age < 30 years [4,12]. While the risk decreases with age, the exact reason is unclear. Angelini has suggested that this may be due to the stiffening of the aortic wall with age, which might help protect against compression of the intramural course [12]. The risk of SCD under the age of 10 is also low [44,54,55]. There is a general consensus among those who have published on this topic that more information is needed to aid in assigning risk [4,12,56]. Recently, the Registry of Anomalous Aortic Origin of the Coronary Artery was established to compare surgical versus observational outcomes and try to identify subsets of patients who would benefit from one approach over the other [57].

### Surgical strategies

Currently, many patients undergo surgery as the primary treatment strategy for this condition. This is particularly true in all cases of ALCA and the symptomatic patient with ARCA and has been supported by a consensus statement in the Guidelines for Management of Adults with Congenital Heart Disease [58].

There are multiple surgical options for treating AAOCA. Bypass grafting was used initially [59,60]. However, early graft failure was reported in several cases. Tavaf-Motamen and colleagues theorized that the early failure is due to steal phenomenon at high levels of exertion [61] or competitive flow from patent native vessels contributing to graft thrombosis [62,63]. For this reason bypass grafting has been used less frequently. Other approaches have included reimplantation of the anomalous vessel into its appropriate sinus [31,64], patch augmentation [65,66] or pulmonary artery translocation to reduce the risk of compression of the anomalous vessel as it traverses between the aorta and the pulmonary artery [67].

More recently, unroofing the anomalous vessel along its intramural segment has become the preferred management option [61,68-70]. This procedure, first reported by Mustafa in 1981, creates a neo-orifice at the anatomically correct sinus [71]. The advantages of unroofing are that it eliminates the intramural segment and avoids an oblique angle of take-off of the vessel. Some surgeons advocate a tailored approach that depends on the specific anatomic variant [66].

The unroofing technique has been highly touted for several years. However, examination of the surgical literature indicates that surgical correction is not without risks. Romp and his colleagues looked at complications associated with the unroofing procedure [70]. The authors prospectively evaluated 9 consecutive patients who underwent surgical repair for anomalous origin of the coronary artery (6 ALCA and 3 ARCA) between 1995 and 2001. Of these 9 patients, 7 underwent an unroofing procedure while 2 had neo-ostial creation without unroofing. Though the authors did not find postoperative ischemia on stress testing, out of the 9 patients studied 1 developed severe aortic insufficiency and required aortic valve replacement.

Brothers and her coworkers, in their 2007 prospective study, reported on myocardial ischemia after surgical repair of an anomalous coronary artery [56]. The authors reviewed the records of 24 patients who underwent surgical correction of anomalous coronary arteries from the opposite sinus (8 ALCA and 16 ARCA) between 2001 and 2006. Preoperatively, of the 24 patients reviewed one patient had a “blunted blood pressure response” on exercise stress test without EKG changes, one had premature ventricular contractions on Holter monitoring and one had an episode of aborted sudden death. Postoperatively, out of the 24 patients, 9 (38%) had evidence of ischemic changes on exercise stress testing, myocardial perfusion scans, or stress echocardiograms that were not seen preoperatively – including 8/16 for ARCA. It should be noted that the same postoperative stress testing regimen had not been uniformly applied preoperatively, making absolute comparisons difficult. These findings are worrisome for the following reasons: 1. If they indicate a false positive, then it makes it difficult to follow these patients after surgery 2. If they are truly an indication of compromised myocardial perfusion, then the operation may be responsible for causing injury in patients who did not have it previously. It is unknown if this operative strategy has altered the natural history of sudden cardiac death, but the results are troubling. The authors concluded only that more information is needed in order to weigh the long-term risks of the anomaly against the long-term risks of surgery.

In a survey conducted by the Anomalous Coronary Artery Working Group [72], post-operative complications included valvular regurgitation, left ventricular dysfunction, chronic pericarditis, pericardial effusion and possible small infarct. In addition, 2 deaths occurred after surgery for this condition: a 7-year-old several weeks after unroofing and a 5-year-old who died within the first postoperative week. No specifics were available on the exact cause of these two deaths. These studies emphasize the infrequent but inherent risks of surgery.

Several authors have examined outcomes after surgical management of AAOCA. These often involve small numbers of patients over a relatively short follow-up period and we therefore summarize the results in Table 3. The majority of the patients in these studies are adults with comorbid conditions that undoubtedly contribute to operative risk. Children would likely fare better, but require coronary patency for several decades.

**Table 3:** Outcomes of surgical management of AAOCA and study limitations

**Author**	**N (ARCA/ALCA)**	**Length of follow-up (mean or median in years)**	**Adverse outcome reported**	**Number of adverse events (ARCA/ ALCA)**	**Study limitations**
Romp et al., 2003 [70]	9 (3/6)	2.4	Valvular dysfunction	1 (0/1)	- Short follow-up
Garcia-Rinaldi et al., 2004 [73]	16 (16/0)	6.8	Graft bleed, vessel occlusion	2 (2/0)	- All adult patients (28-79yo)
					- No postoperative stress testing reported
Erez et al., 2006 [32]	9 (5/4)	1	None	0 (0/0)	- Short follow-up
Alphonso et al., 2007 [74]	4 (2/2)	1.9	None	0 (0/0)	- Short follow-up
					- Small N
Brothers et al., 2007 [56]	24 (16/8)	1.25	Ischemic changes, pericardial effusion	10 (8/2)	- Short follow-up
					- No uniform preoperative testing
Fedoruk et al., 2007 [75]	5 (5/0)	1	RV dysfunction due to air embolus	1 (1/0)	- Short follow-up
					- Small N
Gulati et al., 2007 [33]	18 (10/8)	2.2	Transient complete heart block, worsening heart failure, pericarditis requiring medical treatment	4 (Not specified)	- Short follow-up
					- No postoperative stress testing reported
Hamzeh et al., 2008 [76]	4 (4/0)	2	Distal RCA stenosis requiring stent placement 48 hrs post-op	1 (1/0)	- Short follow-up
					- Small N
					- Reimplantation only
					- All patients #62;30yo
Tavaf-Motamen et al., 2008 [61]	4 (4/0)	0.8	Graft failure	2 (2/0)	- Short follow-up
					- Small N
Davies et al., 2009 [77]	36 (21/15)	1.1	Atrial fibrillation, subdural hematoma (died 2 months later)	7 (not specified)	- Short follow-up
					- No postoperative stress testing reported
elZein et al., 2009 [78]	8 (6/2)	1.2	None	0 (0/0)	- 2 patients lost to follow-up
					- Short follow-up
					- No postoperative stress testing reported
Frommelt et al., 2011 [79]	27 (20/7)	1.8	None	0 (0/0)	- Short follow-up
Krasuski et al., 2011^*^[80]	28 (20/8)	8.2	None	0 (0/0)	- Outcome assessment limited to survival only
Mainwaring et al., 2011 [81]	48 (31/17)	5.7	Pleural effusions, postcardiotomy syndrome, heart block, heart transplantation	8 (not specified)	- No postoperative stress testing reported
Mumtaz et al., 2011 [82]	22 (15/7)	1.4	None	0 (0/0)	- Short follow-up
					- Inconsistent postoperative stress testing

Abbreviations**:** ARCA = Anomalous right coronary artery from the left sinus; ALCA = Anomalous left coronary artery from the right sinus.

^*^ Mortality was the only outcome assessed, all other studies listed reported on morbidity and mortality.

### Non-surgical strategies

A small number of case reports claim successful use of beta-blockers in adults to treat this condition [83-86]. One study treated 56 adult patients with AAOCA and reported no episodes of SCD over a 5-year period [87]. Ouali and his team in Tunisia – where bypass grafting is currently the only surgical option – report that of 16 patients with AAOCA treated with beta-blockers and angiotensin-converting enzyme inhibitors, all are alive and symptom free at 34-month follow-up [88]. Any enthusiasm for the results of these few reports of successful medical management must be tempered by the rarity of SCD, particularly in adults, and over such short follow-up periods.

A large number of individual case reports have been published on the use of stents to manage AAOCA in adults [89-93]. In 2000, Doorey and his colleagues reported on a series of 14 AAOCA patients who were successfully stented [94]. All patients had objective evidence of ischemia that resolved after the intervention. While stenting may have utility, it is difficult to draw conclusions from the scant data available. Further, it has limited application in growing children.

Guidelines in managing this condition focus on the association between SCD and exercise. The American Heart Association /American College of Cardiology (AHA/ACC) have recommended against participation in all competitive sports for patients with AAOCA [95]. To what degree a given activity is thought to comprise a competitive sport is debatable. Certain behaviors are an important part of child development and this is reflected in the recommendation that they participate in physical education classes and other “non-strenuous” physical activities but discourage more rigorous organized team sports [1]. Further, Corrado’s data, discussed above, demonstrates that while the risk of SCD is increased with exercise, there is still a risk of SCD even at rest. No distinction is made in the AHA /ACC guidelines between ALCA and ARCA, despite the knowledge that ALCA is the higher-risk lesion. Twenty-seven percent of respondents to Brothers and her colleagues’ questionnaire on management of AAOCA reported they prevent competitive sports participation but otherwise allow exercise [72].

### Intervention: proceed with caution

Like all management decisions, the advantages of the intervention must be weighed against possible adverse consequences. In the case of AAOCA of either (right or left) origin the data at first glance is compelling for surgical intervention. The perceived “risk” of death still cited by many (but derived from autopsy data) suggests a dire outcome if left untreated. Incidence of SCD in the general population, however, undermines the autopsy data. After our initial cautionary review [1], Gersony followed-up with a compelling argument against surgical management of ARCA in the asymptomatic patient [54]. He cited the low incidence of death from this condition in multiple studies and the risk of ischemia after surgery reported in Brothers and her coworkers’ study [56]. Others have now echoed this position. Lytrivi and her colleagues have questioned the utility of operating on all patients with ARCA *and* ALCA given the rarity of SCD and lack of knowledge on the exact mechanism [96]. Osaki and his group advocate expectant management and frequent reassessment in asymptomatic patients with AAOCA [26]. Cheitlin and MacGregor have argued that the risks of surgery outweigh the risks of SCD in asymptomatic patients with ARCA and therefore advise against it. They also advocate discussing with the patient and family the risks of both SCD and surgery when planning management of the patient with asymptomatic ALCA [97]. These authors are not alone, as nearly twenty-five percent of respondent to Brothers’ recent survey on management of AAOCA do not refer asymptomatic patients for surgery [72].

The comprehensive reviews of sudden death discussed above, as well as those listed in Table 2, have, in our opinion, helped to tip the balance toward observation of the asymptomatic patient.^a^ Further, while the proposed mechanisms by which people with ARCA or ALCA die suddenly are compelling, none are certain. There exists the possibility that surgical correction is not a definitive therapy and these patients may still be at risk of coronary compromise or arrhythmias that short-term studies have not yet captured. In short, surgery may not be the answer to this condition and in operating on all patients with AAOCA we may be doing more harm than good.

#### Suggested management guidelines

Based on the data presented in this paper, we summarize our recommendations as follow:

Symptomatic ARCA or ALCA with evidence of myocardial ischemia: Surgical intervention. Prior to surgical intervention we recommend restriction from competitive sports or other recreational activities that approach the level of exertion seen in organized sports.

Asymptomatic ARCA: We are concerned that the risks of surgery outweigh the potential benefits and therefore advise against surgical intervention, but we recommend restriction from competitive sports or other recreational activities that approach the level of exertion seen in organized sports to further lower the risks. However, individual exceptions may be made if the risk of SCD associated with physical exertion is clear to the patient and that risk is deemed acceptable by both the patient and the cardiologist.

Asymptomatic ALCA: Due to the higher cumulative risk of SCD relative to ARCA in our institution we generally operate on this condition after the age of 10. Prior to surgical intervention, we recommend restriction from competitive sports or other recreational activities that approach the level of exertion seen in organized sports. However, this approach can be tailored to various factors: level of activity of the individual, familial concern and institutional experience with operating on this lesion.

## Summary

The fear associated with AAOCA has driven our reliance on surgery as a primary management approach. We as providers need to have a meaningful discussion with our patients – and ourselves – regarding the benefits and potential consequences of referring all such patients for surgery. Close observation, exercise restriction or medical management may be viable options that are already used by many providers. For some patients, the risk of SCD may be untenable, but we need to present that risk honestly – as far rarer then previously thought.

Frommelt noted recently, “Although surgical intervention for some potentially lethal forms of coronary anomalies is feasible with good early results, no clear best practice has been established for the management of these patients [98].” We hope that this critical analysis of recent data lends further insights to the debate and we look forward to the findings of the newly developed registry studying the natural history and long-term surgical outcomes in AAOCA.

## Endnote

^a^ “Asymptomatic” in our opinion refers to the absence of symptoms specifically caused by myocardial ischemia. We do not consider non-cardiac chest pain a “symptom” – for example, chest pain that is reproduced on exercise stress-testing without objective evidence of ischemia or chest pain that is inconsistent with myocardial ischemia. Syncope (especially exertional) is a far more worrisome symptom unless convincingly vasovagal in origin.

## Abbreviations

AAOCA: Anomalous aortic origin of the coronary artery arising from the opposite sinus; ALCA: Anomalous left coronary artery (arising from the right sinus of Valsalva); ARCA: Anomalous right coronary artery (arising from the left sinus of Valsalva); CT: Computed tomography; IVUS: Intravascular ultrasound; MRA: Magnetic resonance angiography; SCD: Sudden cardiac death; TEE: Transesophageal echocardiography; TTE: Transthoracic echocardiography.

## Competing interests

Josiah M Penalver has no competing interests. Ralph S Mosca has no competing interests. Daniel Weitz has no competing interests. Colin K.L. Phoon has no competing interests.

## Authors’ contributions

JMP worked to review the literature and synthesize that information to formulate the overall argument of the paper. RSM provided a unique perspective as a cardiothoracic surgeon specializing in congenital heart disease. He reviewed the surgical literature and edited the surgical critique. DW reviewed the adult literature and edited those sections dealing with adult management of this condition. CKLP has previously written on this subject and served as overall editor for the paper, helping to review all the available literature and direct the arguments.

## Pre-publication history

The pre-publication history for this paper can be accessed here:

http://www.biomedcentral.com/1471-2261/12/83/prepub
